# Genome Sequences of Three Atypical *Xanthomonas campestris* pv. campestris Strains, CN14, CN15, and CN16

**DOI:** 10.1128/genomeA.00465-13

**Published:** 2013-07-11

**Authors:** Stéphanie Bolot, Brice Roux, Sébastien Carrere, Bo-Le Jiang, Ji-Liang Tang, Matthieu Arlat, Laurent D. Noël

**Affiliations:** INRA, Laboratoire des Interactions Plantes-Microorganismes (LIPM), UMR 441, Castanet-Tolosan, Francea; CNRS, Laboratoire des Interactions Plantes-Microorganismes (LIPM), UMR 2594, Castanet-Tolosan, Franceb; State Key Laboratory for Conservation and Utilization of Subtropical Agro-Bioresources, College of Life, Science and Technology, Guangxi University, Nanning, Guangxi, Chinac; Université de Toulouse, Université Paul Sabatier, Toulouse, Franced

## Abstract

*Xanthomonas campestris* pv. campestris is the causal agent of black rot on *Brassicaceae*. The draft genome sequences of three strains (CN14, CN15, and CN16) that are highly aggressive on *Arabidopsis* have been determined. These genome sequences present an unexpected genomic diversity in *X. campestris* pv. campestris, which will be valuable for comparative analyses.

## GENOME ANNOUNCEMENT

*Xanthomonas campestris* pv. campestris is the causal agent of black rot on a wide range of *Brassicaceae*, including vegetable crops (e.g., cabbages and radish) and weeds (e.g., *Arabidopsis thaliana*) (1). Seven genomic clades (A to G) of *X. campestris* pv. campestris have been defined (2). As yet, genomic resources are available for only clades A and C (3–6). Interestingly, clade G strains seem to represent ancestral *X. campestris* pv. campestris strains that have not been described so far (2).

*X. campestris* pv. campestris CN14, CN15, and CN16 are clade G strains that have been isolated in 2003 in Guilin, China, on *Brassica juncea* var. *foliosa*, *Brassica rapa* subsp. *chinensis*, and *Brassica rapa* subsp. *pekinensis*, respectively (7). These three strains cause typical black rot disease symptoms on cabbages and *Arabidopsis* (2, 7). Shotgun sequencing of genomic DNA was performed on a HiSeq 2000 Illumina platform. For strains CN14, CN15, and CN16, 2,512,066, 12,943,052, and 13,937,309 paired-end reads of 85, 101, and 101 bp were obtained and correspond to 86-, 521-, and 561-fold coverage, respectively. Genome assembly was performed using a combination of SOAP*denovo* (8) and Velvet (9) assemblers and yielded 165, 73, and 80 contigs of >500 bp and an N_50_ of 74,016, 214,266, and 195,559 bp, respectively. The average contig sizes are 30,240, 68,756, and 62,722 bp, the largest being 181,633, 466,491, and 407,991 bp long, for a total genome size of 4,989,674, 5,019,206, and 5,017,785 bp for strains CN14, CN15, and CN16, respectively. Next, 155, 67, and 75 of those contigs were organized into 8, 7, and 7 pseudomolecules, respectively. The largest one corresponds to the chromosome (4,908,255, 4,920,230, and 4,928,563 bp, respectively; 65% G+C content for all) based on *X. campestris* pv. campestris strain 8004 chromosomal organization. Remaining pseudomolecules match to known *Xanthomonas* plasmid sequences and should account for the ca. 20-kb and 50-kb endogenous plasmids found in each strain (2). Genome analysis of the CN14, CN15, and CN16 genomes confirmed the presence of at least 22, 22, and 23 type III-secreted proteins, respectively (2). Due to their highly repetitive nature, transcription activator-like effector sequences could not be assembled and are not represented in the final assemblies. Annotation transfer was performed using RATT (10) with *X. campestris* pv. campestris strains B100, 8004, and ATCC 33913 as references. *De novo* annotation was performed on remaining areas using FrameD (11) and manually inspected. In strains CN14, CN15, and CN16, 4,733, 4,800, and 4,793 coding sequences (CDSs), 53, 52, and 52 tRNA genes, and 3, 4, and 4 rRNA genes could be identified, respectively.

Phylogenetic analyses based on the core genome shared with the 5 *X. campestris* pv. campestris genomes (3–6, 12) and *X. campestris* pv. raphani strain 756C were performed using UNUS (13). This analysis shows that the three clade G strains are most closely related to other *X. campestris* pv. campestris strains but assemble into a novel phylogenetic group of *X. campestris* pv. campestris. Using orthoMCL (percent match cutoff = 80, BLAST parameter F = false) (14), CN14, CN15, and CN16 share 3,635, 3,648, and 3,646 CDSs with the 3 *X. campestris* pv. campestris reference genomes, while Xca5 shares 3,711 CDSs with those three strains (4). In conclusion, the CN14, CN15, and CN16 genomes significantly contribute to our knowledge of intraspecific genomic diversity in *X. campestris* pv. campestris.

### Nucleotide sequence accession numbers.

The annotated whole-genome shotgun sequences of CN14, CN15, and CN16 have been deposited at NCBI under the accession no. AQOP00000000, AQOO00000000, and AQON00000000, respectively.

## References

[1] Vicente JG Holub EB 2013 Xanthomonas campestris pv. campestris (cause of black rot of crucifers) in the genomic era is still a worldwide threat to Brassica crops. Mol. Plant Pathol. 14:2–18 23051837 10.1111/j.1364-3703.2012.00833.x PMC6638727

[2] Guy E Genissel A Hajri A Chabannes M David P Carrere S Lautier M Roux B Boureau T Arlat M Poussier S Noël LD 2013 Natural genetic variation of *Xanthomonas campestris* pv. *campestris* pathogenicity on *Arabidopsis* revealed by association and reverse genetics. mBio 4:e00538-12. 10.1128/mBio.00538-12 23736288 PMC3685212

[3] da Silva AC Ferro JA Reinach FC Farah CS Furlan LR Quaggio RB Monteiro-Vitorello CB Van Sluys MA Almeida NF Alves LM do Amaral AM Bertolini MC Camargo LE Camarotte G Cannavan F Cardozo J Chambergo F Ciapina LP Cicarelli RM Coutinho LL Cursino-Santos JR El-Dorry H Faria JB Ferreira AJ Ferreira RC Ferro MI Formighieri EF Franco MC Greggio CC Gruber A Katsuyama AM Kishi LT Leite RP Lemos EG Lemos MV Locali EC Machado MA Madeira AM Martinez-Rossi NM Martins EC Meidanis J Menck CF Miyaki CY Moon DH Moreira LM Novo MT Okura VK Oliveira MC Oliveira VR Pereira HA 2002 Comparison of the genomes of two *Xanthomonas* pathogens with differing host specificities. Nature 417:459–463 12024217 10.1038/417459a

[4] Bolot S Guy E Carrere S Barbe V Arlat M Noël LD 2013 Genome sequence of *Xanthomonas campestris* pv. campestris strain Xca5. Genome Announc. 1(1):e00032-12. 10.1128/genomeA.00032-12 23405315 PMC3569304

[5] Qian W Jia Y Ren SX He YQ Feng JX Lu LF Sun Q Ying G Tang DJ Tang H Wu W Hao P Wang L Jiang BL Zeng S Gu WY Lu G Rong L Tian Y Yao Z Fu G Chen B Fang R Qiang B Chen Z Zhao GP Tang JL He C 2005 Comparative and functional genomic analyses of the pathogenicity of phytopathogen *Xanthomonas campestris* pv. campestris. Genome Res. 15:757–767 15899963 10.1101/gr.3378705 PMC1142466

[6] Vorhölter FJ Schneiker S Goesmann A Krause L Bekel T Kaiser O Linke B Patschkowski T Rückert C Schmid J Sidhu VK Sieber V Tauch A Watt SA Weisshaar B Becker A Niehaus K Pühler A 2008 The genome of *Xanthomonas campestris* pv. *campestris* B100 and its use for the reconstruction of metabolic pathways involved in xanthan biosynthesis. J. Biotechnol. 134:33–45 18304669 10.1016/j.jbiotec.2007.12.013

[7] He YQ Zhang L Jiang BL Zhang ZC Xu RQ Tang DJ Qin J Jiang W Zhang X Liao J Cao JR Zhang SS Wei ML Liang XX Lu GT Feng JX Chen B Cheng J Tang JL 2007 Comparative and functional genomics reveals genetic diversity and determinants of host specificity among reference strains and a large collection of Chinese isolates of the phytopathogen *Xanthomonas campestris* pv. campestris. Genome Biol. 8:R218. 10.1186/gb-2007-8-6-218 17927820 PMC2246292

[8] Li R Zhu H Ruan J Qian W Fang X Shi Z Li Y Li S Shan G Kristiansen K Li S Yang H Wang J Wang J 2010 De novo assembly of human genomes with massively parallel short read sequencing. Genome Res. 20:265–272 20019144 10.1101/gr.097261.109 PMC2813482

[9] Zerbino DR Birney E 2008 Velvet: algorithms for *de novo* short read assembly using de Bruijn graphs. Genome Res. 18:821–829 18349386 10.1101/gr.074492.107 PMC2336801

[10] Otto TD Dillon GP Degrave WS Berriman M 2011 RATT: rapid annotation transfer tool. Nucleic Acids Res. 39:e57. 10.1093/nar/gkq1268 21306991 PMC3089447

[11] Schiex T Gouzy J Moisan A de Oliveira Y 2003 FrameD: a flexible program for quality check and gene prediction in prokaryotic genomes and noisy matured eukaryotic sequences. Nucleic Acids Res. 31:3738–3741 12824407 10.1093/nar/gkg610 PMC169016

[12] Tao F Wang X Ma C Yang C Tang H Gai Z Xu P 2012 Genome sequence of *Xanthomonas campestris* JX, an industrially productive strain for xanthan gum. J. Bacteriol. 194:4755–4756 22887662 10.1128/JB.00965-12 PMC3415473

[13] Rodriguez-R LM Grajales A Arrieta-Ortiz ML Salazar C Restrepo S Bernal A 2012 Genomes-based phylogeny of the genus *Xanthomonas*. BMC Microbiol. 12:43. 10.1186/1471-2180-12-43 22443110 PMC3359215

[14] Li L Stoeckert CJ Jr Roos DS 2003 OrthoMCL: identification of ortholog groups for eukaryotic genomes. Genome Res. 13:2178–2189 12952885 10.1101/gr.1224503 PMC403725

